# Effect of pH, Temperature, and Chemicals on the Endoglucanases and *β*-Glucosidases from the Thermophilic Fungus* Myceliophthora heterothallica* F.2.1.4. Obtained by Solid-State and Submerged Cultivation

**DOI:** 10.1155/2016/9781216

**Published:** 2016-05-08

**Authors:** Vanessa de Cássia Teixeira da Silva, Amanda Lais de Souza Coto, Rafael de Carvalho Souza, Marcello Bertoldi Sanchez Neves, Eleni Gomes, Gustavo Orlando Bonilla-Rodriguez

**Affiliations:** ^1^Laboratório de Bioquímica de Proteínas, Departamento de Química e Ciências Ambientais, Universidade Estadual Paulista (UNESP), Rua Cristovão Colombo 2265, 15054-000 São José do Rio Preto, SP, Brazil; ^2^Laboratório de Bioquímica e Microbiologia Aplicadas, Departamento de Biologia, Universidade Estadual Paulista (UNESP), Rua Cristovão Colombo 2265, 15054-000 São José do Rio Preto, SP, Brazil

## Abstract

This work reports endoglucanase and beta-glucosidase production by the thermophilic fungus* Myceliophthora heterothallica* in solid-state (SSC) and submerged (SmC) cultivation. Wheat bran and sugarcane bagasse were used for SSC and cardboard for SmC. Highest endoglucanase production in SSC occurred after 192 hours: 1,170.6 ± 0.8 U/g, and in SmC after 168 hours: 2,642 ± 561 U/g. The endoglucanases and beta-glucosidases produced by both cultivation systems showed slight differences concerning their optimal pH and temperature. The number of endoglucanases was also different: six isoforms in SSC and ten in SmC. Endoglucanase activity remained above 50% after incubation between pH 3.0 and 9.0 for 24 h for both cultivation systems. The effect of several chemicals displayed variation between SSC and SmC isoenzymes. Manganese activated the enzymes from SmC but inhibited those from SSC. For *β*-glucosidases, maximum production on SmC was 244 ± 48 U/g after 168 hours using cardboard as carbon source. In SSC maximum production reached 10.9 ± 0.3 U/g after 240 h with 1 : 1 wheat bran and sugarcane bagasse. Manganese exerted a significant activation on SSC *β*-glucosidases, and glucose inhibited the enzymes from both cultivation systems. FeCl_3_ exerted the strongest inhibition for endoglucanases and *β*-glucosidases.

## 1. Introduction

Cellulose is the most abundant polysaccharide in the plant cell wall and consists in a linear chain composed of a varying number of *β*-D-glucopyranose residues, linked by *β*(1 → 4) glycosidic bonds. Cellobiose is considered as the smallest repetitive unit of cellulose and can be hydrolyzed into glucose residues [1, 2]. Cellulose differs from other polysaccharides by its insolubility and a stiff structure naturally resistant to biological degradation, suitable for its structural role in plants [3]. Complete cellulose hydrolysis to glucose demands the synergistic action of endoglucanases, exoglucanases, and *β*-glucosidases. Endo-*β*-1,4-glucanases (EC 3.2.1.4) cleave the amorphous regions of the cellulose chain; exoglucanases or cellobiohydrolases (EC 3.2.1.91) attack the nonreducing or reducing chain ends and *β*-1,4-glucosidases (EC 3.2.1.21) hydrolyze cellobiose releasing glucose [4].

Fungal cellulolytic enzymes have biotechnological applications in food, animal feed, wine, agriculture, biomass refining, and pulp and paper industries [5, 6]. The biggest challenge for the enzymes' biotechnological application is their high cost of production. The search for methodologies that could reduce the cost of enzyme production and microorganisms with high growth rate and are able to develop in cheap and easily accessible carbon sources is the current approach to change this paradigm [7]. Thus, various cellulosic substrates such as sugarcane bagasse, corn stover, wheat straw, and wheat bran have been tested by several authors for production of cellulolytic enzymes [8, 9].

The composition of the medium can influence the profile of enzymatic expression by the microorganism. Cellulases can be obtained more economically by microorganisms through submerged or solid-state cultivation. The low water activity in solid-state cultivation (SSC) shows a simple technique of high productivity, but it can affect the heat transfer to the substrate and thus could affect the growth of the microorganism [10]. The use of thermophilic microorganisms can be an alternative to this technical limitation since they tolerate higher temperatures.

The present study aimed to compare the production of endoglucanases and *β*-glucosidases in solid-state and submerged cultivation media by thermophilic* Myceliophthora heterothallica* and to characterize the crude extracts obtained from both systems.

## 2. Materials and Methods

### 2.1. Microorganism

The thermophilic fungus* Myceliophthora heterothallica* F.2.1.4. was isolated from sugar cane bagasse compost and maintained at the Laboratory of Applied Biochemistry and Microbiology, UNESP.

### 2.2. Inoculum Preparation

The inoculum was prepared by growing the fungus* M*.* heterothallica* F.2.1.4. in 250 mL Erlenmeyer flasks with 50 mL medium composed of 1.0% sugarcane bagasse (ground to 1-2 mm); 0.14% (NH_4_)_2_SO_4_; 0.20% KH_2_PO_4_; 0.03% CaCl_2_; 0.02% MgSO_2_·7H_2_O; 0.50% beef peptone; 0.20% yeast extract; 0.03% urea; 0.10% micronutrients solution (5 mg/L FeSO_4_·7H_2_O; 1.6 mg/L MnSO_4_·H_2_O; 1.4 mg/L ZnSO_4_·7H_2_O; and 2.0 mg/L CoCl_2_); 0.2% glucose; and 2.0% agar, pH 5.0. All the solutions and the culture media were sterilized by autoclaving at 121°C for 20 minutes. This medium was inoculated with suspension obtained scrapping the mycelium from culture grown for 3 days at 45°C in 150 mL of nutrient solution (g/L): 3.5 (NH_4_)_2_SO_4_; 3.0 KH_2_PO_4_; 0.5 MgSO_4_·7H_2_O; 0.5 CaCl_2_; and 0.1% v/v Tween 80, pH 5.0.

### 2.3. Submerged Cultivation (SmC)

Endoglucanase and *β*-glucosidase production in SmC was carried out in 250 mL Erlenmeyer flasks using cardboard as a carbon source, previously cut and ground in a blender. The mixture contained 1% cardboard and 20 mL of the nutrient solution described above was inoculated with 2 mL of the microorganism inoculum as described. The cultures were incubated on a rotary shaker (100 RPM) at 45°C for 10 days and samples were taken every 24 h, filtered through vacuum using Whatman paper number 1, and centrifuged at 6,000 ×g for 20 minutes. The supernatant was used as a crude enzyme solution.

In order to evaluate the use of agroindustrial residues such as wheat bran and sugar cane bagasse as the substrate in SmC, they were washed, ground, and oven-dried (60°C for 48 h). The sugarcane bagasse was sieved, allowing selecting particles ranging from 1 to 2 mm. Subsequently, cultivation was carried out in 250 mL Erlenmeyer flasks containing 1% (1 : 1 w/w) of wheat bran and sugarcane bagasse, 20 mL nutrient solution, and 2 mL inoculum suspension. The medium was previously autoclaved as described. The cultures were incubated on a rotary shaker (100 RPM) at 45°C for 10 days and sampling was performed as explained above.

### 2.4. Solid-State Cultivation (SSC)

The substrates used in the SSC were composed of used 5 grams of cardboard or a mixture of wheat bran and sugar cane bagasse (1 : 1). The substrates were inserted in polypropylene bags (12 × 20 cm) and sterilized at 121°C for 40 min. The fungus inoculation was carried out by adding 20 mL of the mycelium suspension. The moisture content of around 80% was reached with the addition of sterile distilled water. The fermentation was at 45°C for 14 days. Every 48 h, one bag was taken and the fermented material was mixed with 20 mL of distilled water per gram, stirred for 30 min, filtered, and centrifuged at 10,000 ×g, at 10°C. The supernatant was used as a crude enzyme solution.

### 2.5. Enzymatic Assay

Endoglucanase activity was measured at 60°C in a reactional mixture composed of 0.9 mL of 100 mM L^−1^ sodium acetate buffer pH 5.0 containing 4% of carboxymethyl cellulose (CMC) (Sigma-Aldrich, USA) and 0.1 mL of crude enzyme solution. The reducing groups, expressed as glucose, released were assayed by using the DNS (3,5-dinitrosalicylic acid) method [11]. One unit of endoglucanase activity was defined as the amount of enzyme able to release 1 *μ*mol of reducing sugars per min under the assay conditions [12].

*β*-D-Glucosidase activity was measured in a mixture of 50 mm sodium acetate buffer, pH 5.0, 500 *μ*L of 2 mM L^−1^ p-nitrophenyl-*β*-D-glucopyranoside (pNP-Glc) as substrate, and 50 *μ*L of the enzyme solution incubated at 60°C for 10 min. The reaction was stopped by addition of 2 mL of 2 M Na_2_CO_3_ and the absorbance was read at 410 nm. One unit of *β*-glucosidase was defined as the amount of enzyme that releases 1 *μ*mole of nitrophenol/min in the reaction conditions.

All the enzyme assays were performed in triplicate.

### 2.6. Zymogram for Enzyme Activity

The endoglucanase solution was analyzed by electrophoresis on denaturing 10% sodium dodecyl sulphate (SDS) polyacrylamide gel using Tris-Glycine buffer pH 8.3. After electrophoresis, the enzyme was renatured with 1% triton X-100 for 30 minutes and subsequently the gel was overlaid with a mixture of 1% (w/v) CMC (Sigma-Aldrich) as substrate and 1% (w/v) agarose and incubated for 30 min at 50°C. For detection of endoglucanase activity, the gel was immersed in a 0.1% (w/v) Congo Red solution for 15 minutes and then washed with 1 M L^−1^ NaCl until visualization of the bright spots corresponding to the enzyme [13].

For *β*-glucosidase, the gel was incubated with 0.2 M L^−1^ acetate buffer pH 5.0 for 10 minutes at room temperature under gentle agitation. After that, the gel was treated with a solution of esculin 0.1% ferric chloride in 0.03% 0.2 M L^−1^ acetate buffer pH 5.0 until the appearance of bands [14].

### 2.7. Enzyme Characterization

The effect of pH on the enzyme activity was evaluated as described above, in the pH range from 2.0 to 11.0 using 0.1 M buffers: pH 2-3: sodium citrate; pH 3–5.5: sodium acetate; pH 6.0–7.5: HEPES; pH 8.0–9.5: glycine; and pH 10.0–11.0: CAPS. The effect of temperature was assayed incubating the reactional mixture in temperature range from 35 to 85°C in the optimal pH.

The effect of pH on enzyme stability was studied by incubating the crude enzyme solution in various buffers with pH ranging from 2.5 to 11.0 during 24 h at 25°C, followed by the determination of endoglucanase or *β*-glucosidase residual activity. Thermal stability was determined by incubating the crude enzyme from 40 to 70°C for 60 min, and residual activity was assayed as described above. For both experiments the data were compared to a control without preincubation, denoted as 100%.

### 2.8. Effect of Different Compounds on Endoglucanase/*β*-Glucosidase Activity

The enzyme activity assays were done in the presence of metal ions and other compounds: ethanol, glucose, triton, and metal ions. The metal ions (chloride salts) were of analytical grade and were dissolved in ultrapure water (UPW). The extract was dialyzed against UPW before the assays. The results were compared by Student's *t*-test for independent samples using BioEstat 5.3 [15].

## 3. Results and Discussion

### 3.1. Endoglucanase Production by SSC and SmC

**Figure 1:**
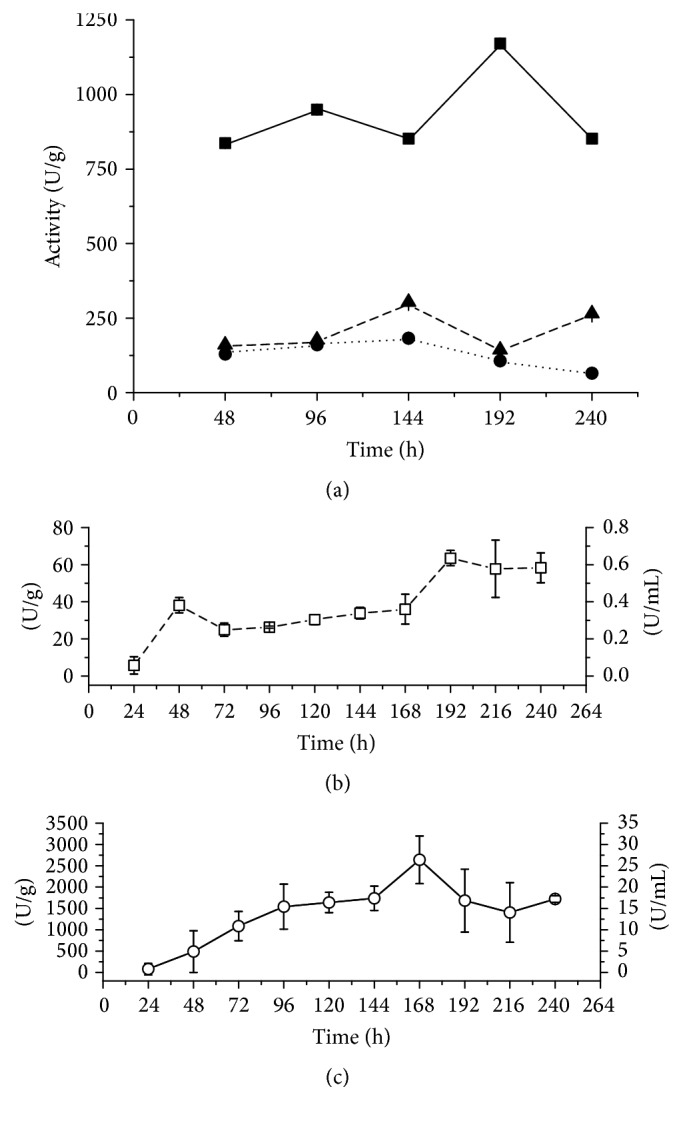
(a) Endoglucanase production (expressed as U/g) by* M*.* heterothallica* F.2.1.4. in solid-state cultivation. (■) SSCWB; (●) SSCP; (▲) SSCLB. Endoglucanase production in submerged cultivation expressed as U/g and U/mL. (b) (□) SmCWB; (c) (○) SmCP.

The cultivation of* Myceliophthora heterothallica* by SSC and SmC using different lignocellulosic residues as substrates revealed that the extracellular enzyme production was highly dependent on the chemical composition of the tested culture media (wheat bran, sugarcane bagasse, and cardboard). When the ratio of 9 : 1 sugarcane bagasse/wheat bran (SSCSB) (5 g of total carbon source) was used, endoglucanase activity was 303.0 ± 1.7 U/g after 144 hours, and the production in SSC using cardboard (SSCP) achieved 182.7 ± 1.6 U/g. Changing the ratio of sugarcane bagasse/wheat bran to 1 : 1 (SSCWB), the production rose more than three times to 1,170.6 ± 0.8 U/g after 192 h (Figure 1(a)). The values found for SSCSB agree with those reported for* Aspergillus terreus* [16]. Another fungus,* Fusarium oxysporum,* showed a similar production [17]. High yields as those found by SSCP in this work were also shown by the mutant* Penicillium janthinellum* EU2D21 [18].

For SmC (Figure 1(b)), cardboard or wheat bran in mixture with sugarcane bagasse at 1 : 1, in concentration of 1% w/v (0.4 g of total carbon source), was used. In medium with wheat bran the lowest production was obtained, 63.5 ± 4.1 U/g, after 192 h, and using cardboard (SmCP) the endoglucanase production was increased to 2,642 ± 561 U/g, in 168 h (Figure 1(c)).

Wheat bran is the most common substrate for solid cultivation since it is rich in proteins, carbohydrates, and minerals that promote supplying macro- and micronutrients for microbial growth [19, 20]. The sugarcane bagasse is composed of cellulose (44%), hemicellulose (25–27%), lignin (20–22%), ashes, and minerals [21]. The addition of wheat bran to sugarcane bagasse can supply nutritional condition for growth and enzyme production in SSC. On the other hand, cardboard is the substrate with the highest percentage of cellulose, 63 ± 1.6%, and also 14% hemicellulose and less than 5% of lignin [22]. This lower lignin content makes the cellulose more accessible to the microorganism and could improve the liberation of cellulase inducers.

The analysis of enzyme by zymography showed different profiles for the endoglucanases according to the system and carbon source used in the cultivation (Figure 3(a)). For the ratio (1 : 1) of sugarcane bagasse and wheat bran in SSC and SmC six isoenzymes were observed whose molecular masses follow around 116, 66, and 30 kDa. The middle range isoenzymes have faded bands. This profile was modified when using cardboard as the carbon source; the same six isoenzymes previously mentioned were also expressed in SSC, but the 66 kDa bands displayed higher activity and four other isoforms with intermediary molecular weight appeared clearly in submerged cultivation, suggesting that the higher accessibility of cellulose from cardboard in SmC may have influenced the production of more isoenzymes to facilitate digestion. The profile for the SSC using cardboard did not display a defined profile but also suggests the expression of intermediate molecular weight isoforms.

### 3.2. *β*-Glucosidase Production by SSC and SmC

**Figure 2:**
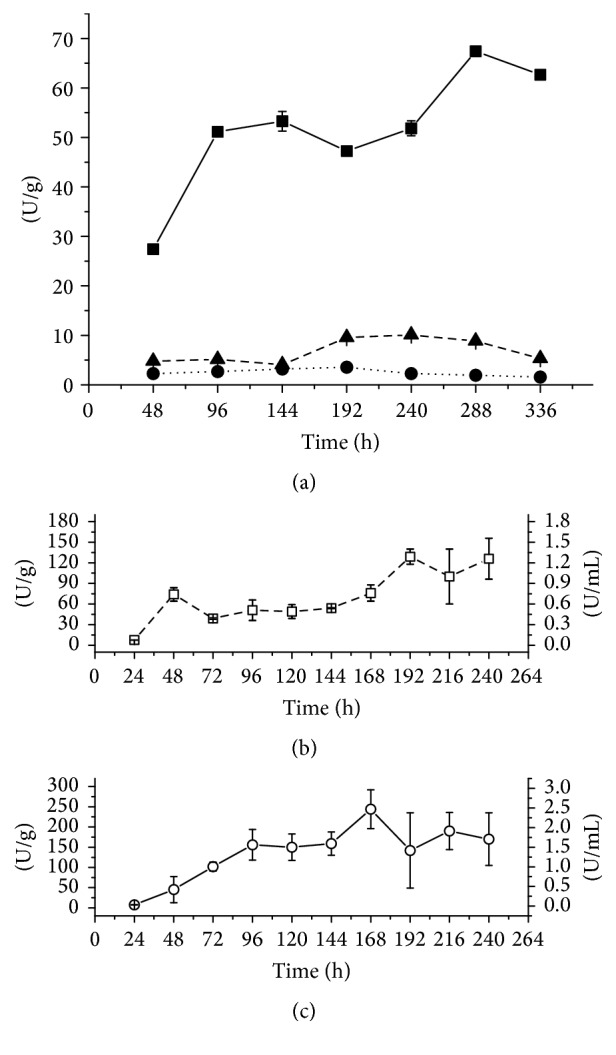
(a) *β*-Glucosidase production (expressed as U/g) by* M*.* heterothallica* F.2.1.4. in solid-state cultivation. (■) SSCWB; (●) SSCP; (▲) SSCLB. *β*-Glucosidase production in submerged cultivation expressed as U/g and U/mL. (b) (□) SmCWB; (c) (○) SmCP.

The peak of enzyme production by SSC was obtained after 192 hours of cultivation. When the 9 : 1 ratio of sugarcane bagasse (SSCSB) (5 g of carbon source in total) was used, *β*-glucosidase activity was 10.9 ± 0.3 U/g after 240 h, and replacing the proportion of sugarcane bagasse with 1 : 1 (SSCWB), production increased to 67.3 ± 0.8 U/g after 288 h (Figure 2(a)). When using cardboard in SSC, the production decreased to 3.62 ± 0.01 U/g. The fungus* Fomitopsis* sp. RCK2010 produced 53.68 U/g using only wheat bran, and* Thermoascus aurantiacus* produced 48 U/g with the same substrate [8, 23]. On the other hand, using SmC (Figure 2(b)), only two types of substrates were compared (1% of total carbon source, 0.4 grams): cardboard and wheat bran with sugarcane bagasse (1 : 1) (SmCWB). Under these experimental conditions, the lowest production 129 ± 11 U/g was obtained after 192 hours, but using cardboard (SmCP) production almost doubled to 244 ± 48 U/g after 168 hours of cultivation (Figure 2(c)).

**Figure 3:**
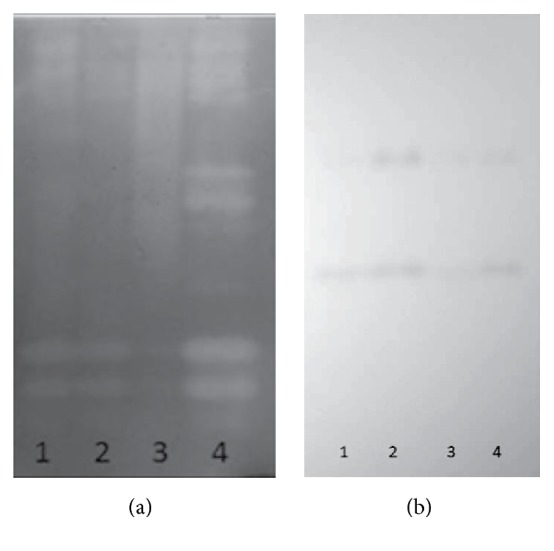
Expression profile analyzed by zymography: (a) endoglucanase isoforms produced by* M*.* heterothallica* F.2.1.4. in submerged (Sm) and solid-state (SS) cultivation. Line 1, SSCWB; Line 2, SmCWB; Line 3, SSCP; and Line 4, SmCP; (b) *β*-glucosidases produced by submerged and solid-state cultivation. Line 1, SSCWB; Line 2, SmCWB; Line 3, SSCP; and Line 4, SmCP.

The zymogram of beta-glucosidases (Figure 3(b)) showed different expression levels according to the cultivation system and the carbon source used. In all cultivation systems only two isozymes around 146 and 66 kDa were produced. Apparently the use of the ratio (1 : 1) isoenzyme lower molecular weight had increased expression.

### 3.3. Comparison of the Results Obtained by Both Cultivation Systems

Although by submerged cultivation (SmC) higher values of productivity were obtained in terms of U/g compared to cultivation in the solid state, two aspects must be considered: first, the volume of liquid obtained in the process. In SmC, the enzyme was diluted 100 times while in SSC it was diluted only 20 times. Since the aim of this project was the production of enzymes for use in their crude form, the initial concentration of the solution is important because it avoids a step to concentrate the enzyme, reducing the process costs and showing a frequent decreased yield of the enzyme due to nonspecific adsorption to membranes and concentrators, for example, an advantage of SSC.

On the other hand, previous data from our group (unpublished) showed no linearity between the increase in concentration of the substrate (C source) from 1 to 2.5% and enzyme production, a fact also described by Kumar et al. [24] working with* Aspergillus niger*, who in fact verified a decrease in cellulase and pectinase production above a certain concentration of the carbon source.

In addition, the cultivation system also influences enzyme production since SSC has absence of water among the particles of the solid substrate which have been reported as an important factor that interferes in the extracellular enzymes secretion [25]. The endoglucanase production in SSC has the best benefit-cost ratio, since it allows using agroindustrial residues, resembles the environmental conditions found by the fungus, has high productivity, and demands a small area for production [10].

### 3.4. Crude Enzyme Characterization

#### 3.4.1. Endoglucanases

**Figure 4:**
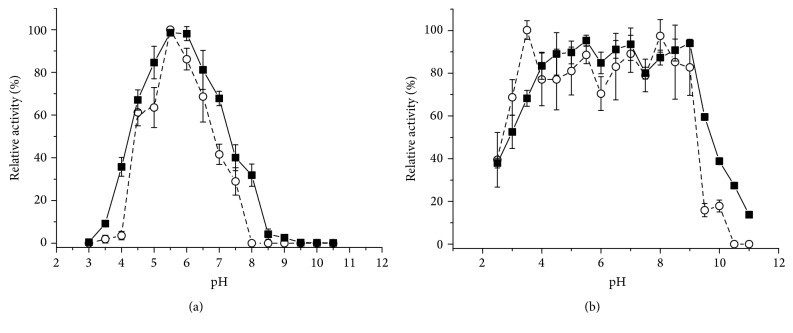
Effect of pH on the activity of the endoglucanases from the crude extract from* M*.* heterothallica* F.2.1.4. (a) (■) SSCWB and (○) SmCP, respectively. Effect of the pH on the stability of the endoglucanases. (b) (■) SSCWB and (○) SmCP, respectively.

A biochemical characterization was carried out for the highest SSC and SmC productions, namely, SSCWB and SmCP, respectively. The effect of pH on the endoglucanase activity was studied using CMC (4%) as substrate, and the endoglucanases produced by both systems showed maximum activity at pH 5.5 (Figure 4(a)).

Reported values have been found for optimum pH in the crude extract in the range from pH 3.5 to 6.0, independent of the cultivation system [26, 27]. The crude endoglucanase solution obtained from* Pycnoporus sanguineus* in SmC showed optimum pH around 3.5–4.0 [27]. Similarly, [28, 29] obtained the maximum activity at pH 4.0 for endoglucanases from* Trichoderma atroviride* 676 and* Penicillium* sp. CR-316, respectively, cultivated in SmC, while others found a higher range, from 4.5 to pH 6.0, for the endoglucanases from the fungus* Acremonium* sp. produced in SSC [26], and those from* M*.* thermophila* M.7.7 exhibited an optimum pH of 5.0 [30]. The endoglucanases produced by* A*.* niger* in SSC exhibited higher activity at pH 4.0 [31, 32].

The effect of the pH on the stability (Figure 4(b)) was analyzed in the pH range from 2.5 to 11 after incubation for 24 hours at room temperature (25°C). Endoglucanases produced by SSC and SmC exhibited high activity above pH 3.5–4.0, up to pH 9.0. The residual activity falls sharply at more acid or alkaline pH values.

**Figure 5:**
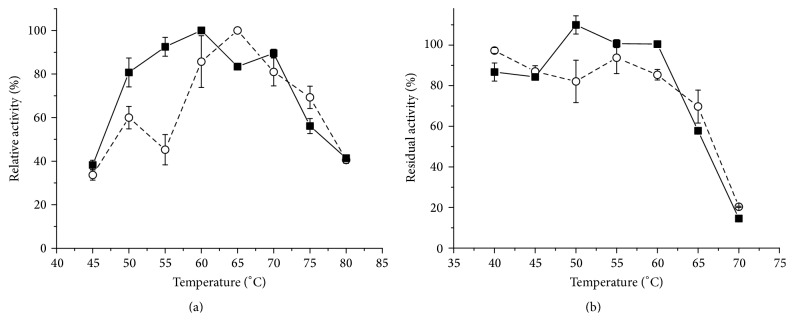
Effect of temperature on the activity of endoglucanase of the crude extract from* M*.* heterothallica* F.2.1.4. at pH 5.5. (a) (■) SSCWB and (○) SmCP, respectively. Temperature effect on the stability of the endoglucanases. (b) (■) SSCWB and (○) SmCP, respectively.

The effect of temperature on endoglucanase activity (Figure 5(a)) was analyzed at pH 5.5 for the extracts obtained in both cultivation systems. The profiles are uneven, suggesting that the isoforms have different values for the temperature optima. For enzyme from SSC, the maximum activity was reached at 60°C, which decreased to 40% at 80°C. On the other hand, for the enzymes obtained in SmC, the optimum temperature was 65°C, the same value found for endoglucanases obtained in SSC from* A*.* niger* [32]. Endoglucanases from* A*.* niger* obtained by SSC have an optimum temperature of 60°C [31], and those obtained by SSC from* M*.* thermophila* M.7.7 exhibited maximum activity at 70°C [30].

On the other hand, analyzing the enzymes obtained by SmC, those of* P*.* sanguineus* displayed an optimum temperature of 60°C [27] and values between 60 and 70°C for the endoglucanases from* T*.* atroviride* 676 [28] and similar values were reported for the enzymes from the fungus* Penicillium* sp. CR-316 [29].

Concerning the thermal stability of endoglucanases (Figure 5(b)), the residual activity was measured after incubation of the extract at different temperatures, between 40 and 70°C, for 1 hour. The endoglucanases produced by SmC displayed activity above 80% between 40 and 60°C. On the other hand, the endoglucanases produced by SSC exceed 100% of residual activity at 50°C and only decay after 60°C. Endoglucanases produced by SSC and SmC lost around 80% of their activity during 1 hour at 70°C. These endoglucanases were more stable than those produced by SSC from* M*.* thermophila* M.7.7 [30].

The effect of several compounds on endoglucanase activity was analyzed using the crude extract produced by SSCWB and SmCP (Table 1). The effects showed some discrepancies between the enzymes produced in both conditions, showing opposite effects or different degrees of activation/inhibition. The most remarkable difference occurred for MnCl_2_, which caused a strong inhibition for the endoglucanases obtained by SSC, while increasing the activity of the enzymes from SmC, suggesting that the “new” isoenzymes expressed in this cultivation system and described above would be significantly activated by that cation. Since EDTA inhibited the endoglucanases produced by both cultivation systems, it was expected that a divalent cation would increase the enzyme activity. Iron inhibition could be attributed to the effect of Fe^3+^ at the reducing ends of cellulose [33].

#### 3.4.2. *β*-Glucosidases

**Figure 6:**
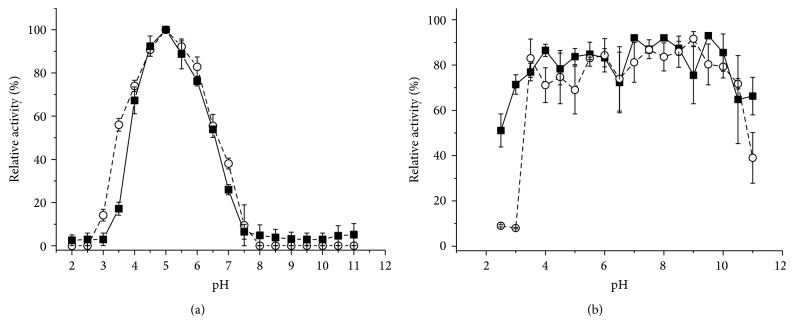
Effect of pH on the activity of the *β*-glucosidases from the crude extract from* M*.* heterothallica* F.2.1.4. (a) (■) SSCWB and (○) SmCP, respectively. Effect of the pH on the stability of the *β*-glucosidases. (b) (■) SSCWB and (○) SmCP, respectively.

The *β*-glucosidase activity produced by both cultivation systems showed maximum activity at pH 5.0 (Figure 6(a)). Reported values for optimum pH of fungal *β*-glucosidases fall into a wide range, 2–2.5 [34], 4.5 [23, 27], 4.8 [35], and 6.0 [36], but most of these enzymes display higher activity in the pH range 4-5 [37]. The effect of the pH on the stability (Figure 6(b)) was analyzed on the pH range from 2.5 to 11 after incubation for 24 hours at room temperature (25°C). The *β*-glucosidases produced by SSC and SmC exhibited high activity above pH 3.5, up to 10.0. The residual activity falls sharply at more acid or alkaline pH values.

**Figure 7:**
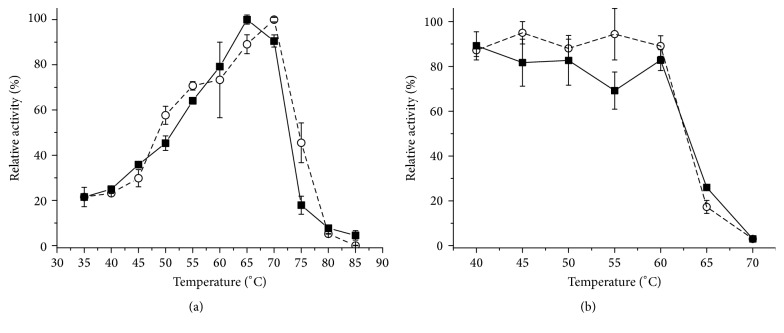
Effect of temperature on the activity of *β*-glucosidase of the crude extract from* M*.* heterothallica* F.2.1.4. at pH 5.0. (a) (■) SSCWB and (○) SmCP. Temperature effect on the stability of the *β*-glucosidases. (b) (■) SSCWB and (○) SmCP.

The effect of temperature on *β*-glucosidase activity is shown in Figure 7(a), analyzed at pH 5.0 for the extracts obtained in both cultivation systems. The profile for SmC suggests that the isoforms have different values for the temperature optima. For SSC, the maximum value was 65°C and for SmC 70°C. Falkoski et al. [27] found an optimum temperature of 55°C and for Iembo et al. [34] it was 65°C.

The thermal stability (Figure 7(b)) was determined by measuring residual activity after incubation at 40–70°C during 1 hour. Both crude extracts were stable up to 60°C.

The effect of several compounds on *β*-glucosidase activity was analyzed using the crude extract produced by SSCWB and SmCP (Table 1). The effects showed some discrepancies between the enzymes produced in both conditions, showing opposite effects or different degrees of activation/inhibition. The most remarkable difference occurred for MnCl_2_, which caused a strong activation for *β*-glucosidase obtained by SSC, while increasing the activity of those enzymes from SmC.

## 4. Conclusions

The thermophilic fungus* M*.* heterothallica* F.2.1.4. proved to be a great producer of endoglucanases, using both sugarcane bagasse and wheat bran by solid-state cultivation or using cardboard in submerged cultivation. Cardboard in SmC would be a residue of easy access and would be able to induce the synthesis of more isoforms of endoglucanases, which, when characterized in the crude extract, was shown to be thermostable and prompted us to perform further studies of biotechnological applications. The beta-glucosidases obtained by SmC showed higher stability.

## Figures and Tables

**Table 1:** Effect of different compounds on endoglucanase and beta-glucosidase relative activity of the dialyzed crude extract expressed as mean values ± SD. NT = not tested; ND = not detected. The asterisks represent significant differences against the control with *p* < 0.05 according to Student's *t*-test for means.

Compound	Conc. (mM)	Endoglucanases		*β*-Glucosidases	
		Rel. act. SSC (%)	Rel. act. SmC (%)	Rel. act. SSC (%)	Rel. act. SmC (%)
PVA	10	120.9 ± 2.8^*∗*^	110.2 ± 10.4	NT	NT
Isopropanol	10	116.7 ± 0.7^*∗*^	74.1 ± 1.6^*∗*^	106.4 ± 4.1	92.9 ± 5.0
NaCl	10	113.3 ± 0.7^*∗*^	90.3 ± 3.9^*∗*^	95.9 ± 3.9	71.5 ± 2.5^*∗*^
DTT	10	112.5 ± 0.9^*∗*^	121.4 ± 4.9^*∗*^	86.4 ± 2.6^*∗*^	92.9 ± 3.6
PMSF	1	110.2 ± 0.3^*∗*^	110.2 ± 11.2	116.4 ± 4.8^*∗*^	101.2 ± 6.4
Glucose	10	109.5 ± 0.9^*∗*^	89.2 ± 12.5	59.4 ± 6.1^*∗*^	69.0 ± 1.1^*∗*^
DMSO	10	109.3 ± 2.3^*∗*^	108.9 ± 18.8	NT	NT
Acetone	10	101.0 ± 1.2	95.9 ± 8.4	92.7 ± 3.4	106.1 ± 5.4
Triton X-100	10	101.2 ± 1.3	114.3 ± 16.7	NT	NT
Ethanol	10	101.0 ± 2.9	71.4 ± 3.7^*∗*^	94.7 ± 11.2	85.0 ± 5.3
MgCl_2_	10	89.7 ± 2.1^*∗*^	35.1 ± 3.4^*∗*^	99.3 ± 3.6	76.7 ± 1.0^*∗*^
PEG 8000	3	85.5 ± 0.4^*∗*^	93.7 ± 5.3	NT	NT
PEG 3350	10	112.8 ± 1.7^*∗*^	97.1 ± 7.6	NT	NT
CaCl_2_	10	68.3 ± 1.0^*∗*^	31.4 ± 2.5^*∗*^	79.5 ± 7.6^*∗*^	100.7 ± 2.0
SDS	10	57.0 ± 0.8^*∗*^	82.1 ± 4.5^*∗*^	79.9 ± 1.6^*∗*^	80.8 ± 2.9^*∗*^
EDTA	10	84.5 ± 2.7^*∗*^	74.8 ± 13.1^*∗*^	89.2 ± 8.9	94.0 ± 2.5
AlCl_3_	10	30.5 ± 0.5^*∗*^	1.7 ± 0.7^*∗*^	26.9 ± 10.7^*∗*^	21.2 ± 0.7^*∗*^
MnCl_2_	10	14.2 ± 1.4^*∗*^	113.0 ± 3.1^*∗*^	177.0 ± 11.7^*∗*^	105.0 ± 1.7
FeCl_3_	10	11.0 ± 0.4^*∗*^	ND^*∗*^	21.3 ± 14.1^*∗*^	ND^*∗*^
